# Mouse Phenome Database

**DOI:** 10.1093/nar/gkt1159

**Published:** 2013-11-15

**Authors:** Stephen C. Grubb, Carol J. Bult, Molly A. Bogue

**Affiliations:** The Jackson Laboratory, 600 Main Street, Bar Harbor, ME 04609 USA

## Abstract

The Mouse Phenome Database (MPD; phenome.jax.org) was launched in 2001 as the data coordination center for the international Mouse Phenome Project. MPD integrates quantitative phenotype, gene expression and genotype data into a common annotated framework to facilitate query and analysis. MPD contains >3500 phenotype measurements or traits relevant to human health, including cancer, aging, cardiovascular disorders, obesity, infectious disease susceptibility, blood disorders, neurosensory disorders, drug addiction and toxicity. Since our 2012 NAR report, we have added >70 new data sets, including data from Collaborative Cross lines and Diversity Outbred mice. During this time we have completely revamped our homepage, improved search and navigational aspects of the MPD application, developed several web-enabled data analysis and visualization tools, annotated phenotype data to public ontologies, developed an ontology browser and released new single nucleotide polymorphism query functionality with much higher density coverage than before. Here, we summarize recent data acquisitions and describe our latest improvements.

## INTRODUCTION

The importance of animal models, and in particular the mouse, has been firmly established for basic and translational research. The laboratory mouse is especially powerful because thousands of inbred and genetically modified strains are currently available and more are being created and phenotyped (1,2); there is a reference genome and several mouse strains are fully sequenced (3,4); commercial genotyping arrays are available; experimental conditions can be precisely controlled; and defined interventions can be performed that cannot be ethically or practically performed on humans.

The past several decades of scientific literature are replete with experiments and findings involving various genetically diverse strains of mice. Phenotype measurements are summarized in publications; however, the primary data are often not typically published or easily accessible. The availability of well-curated standardized measurement data is essential for integrative studies and systems genetics. To support these research efforts, we collect and integrate mouse strain survey data procured from public databases or contributed by members of the scientific community. The Mouse Phenome Database (MPD; phenome.jax.org) serves as a central data repository and houses quantitative phenotype data, gene expression data and genotype data. Data analysis and visualization tools are provided through a web interface. Protocols, experimental conditions and animal environmental history accompany each data set.

Convenient access to annotated and standardized strain data provides essential baseline information and enables investigators to choose appropriate strains for many research applications. For example, a researcher interested in a classical inbred strain such as DBA/2J can use MPD to find typical DBA/2J values for body weight, cholesterol, heart rate or many other phenotypes; to see how DBA/2J responds to alcohol, cocaine or a high-fat diet; to determine the DBA/2J genotype in a region of interest; or to find gene expression probesets where DBA/2J is an outlier.

Our primary activity has been collecting mouse strain survey data sets, where mice from 10 to 40 (or more) strains are tested following a defined protocol under controlled conditions, females and males analyzed separately, with sample sizes as high as feasible. As MPD’s original focus was on collecting baseline measurement data for inbred strains, the database does not yet contain a significant amount of quantitative data on individual mutants, other manipulated lines, or outbred mice. However, we have shifted data acquisition priorities to track with research trends in the community and are starting to accommodate phenotype data from these strain types and emerging panels such as Collaborative Cross (CC) lines and Diversity Outbred (DO) populations. Another vital part of our mission is to provide a home base for data sets supported by investments made by the NIH and other funding sources. Over 270 investigators from 14 countries have contributed data and are supported by ∼130 funding agencies and research foundations worldwide, including all institutes of the NIH. In this manuscript, we present a status report of our current data content, review updates and improvements made since our last NAR update (5) and discuss future plans.

## CONTENTS

MPD current contents are shown in Table 1. MPD contains baseline, intervention and aging data for >200 projects involving several genetic reference populations, including inbred, recombinant inbred (RI), chromosome substitution strains, Hybrid Mouse Diversity Panel (HMDP), F1 hybrids, Collaborative Cross lines (CC) and Diversity Outbred mice (DO). The HMDP is a large panel of ∼100 strains (30 classical inbred, 70 RI) (6,7). CC lines are RI strains derived from eight founder inbred strains (including three wild-derived inbred strains) (8,9). DO mice are an outbred population, derived from incipient CC (pre-CC) lines at early stages of breeding (10–12). At the request of the community, MPD is accessioning data from these new strain panels, which are powerful research tools for complex trait analysis and high-resolution mapping (13).

**Table 1.** MPD current contents

Strains	1330+
Collaborative Cross (emerging lines)	35%
Recombinant inbred lines	25%
Inbred strains	23%
F1 hybrids (including CC diallel)	10%
Chromosome substitution strains	4%
Other	3%
Diversity Outbred population (number of mice)	[283]
Phenotype (200+projects)	3500+
Baseline measurements	68%
Treatment and intervention measurements	32%
Gene Expression (13 sources)	
Number of distinct probe IDs (thousand)	125+ K
Number of data points (million)	∼12 M
Genotype (18 data sets)	
SNP calls (billion)	1.8+ B
Indel calls (million)	18+ M
Structural variant calls (thousand)	∼600 K
Total genome-wide locations represented (million)	60+ M

Over 1330 strains of mice are represented in MPD where phenotypic, gene expression or genotypic data are available. Table 1 shows the percentage of each strain type (count does not include DO mice). There are significantly more strains than previously reported (at 750 strains) because of the recent inclusion of data from pre-CC lines. MPD houses data for >3500 phenotype measurements. MPD’s genotype database is a consolidation of data from 18 community sources containing strain calls for 1.8 + billion single nucleotide polymorphisms (SNPs), 18 + million indels and 600 000 structural variants. Genotypic variation is represented at 60 + million genome-wide locations. The MPD gene expression sector contains ∼12 million mean data points from 13 projects, representing 125 000 probesets.

In addition to strain data, MPD houses an extensive library of detailed validated protocols, which users can access and use to compare their own experimental results. Most data sets are directly associated with a peer-reviewed publication, providing an important layer of data validation.

## DATA UPDATES

### Phenotype

**Figure 1.**
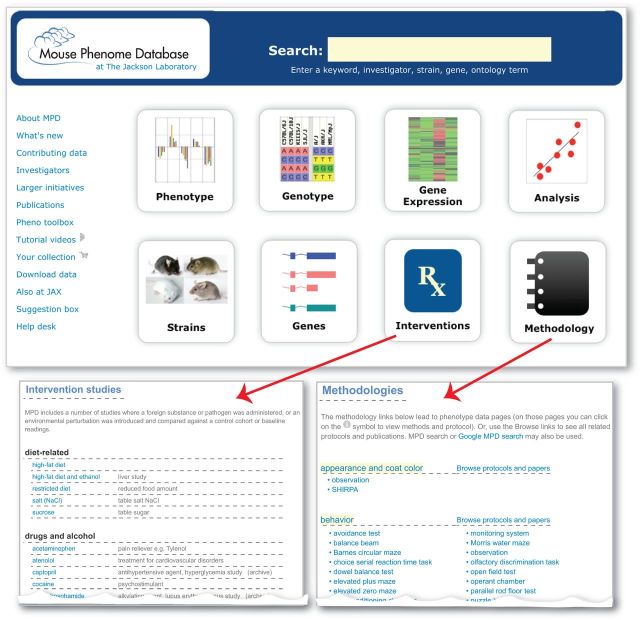
Improved intervention and methodology classification and browsing. The interventions page has been restructured, and trade name equivalents added for all drugs to our existing catalog of synonyms. Interventions are grouped in these sections: diet-related, drugs and alcohol, exercise, pathogens/parasites and toxicity/challenges/mutagens. Measurements have been annotated to a controlled vocabulary of methodology (apparatus, platform). This was done with the recognition that sufficiently classifying behavioral data is more complicated than other phenotypic domains (some users search on apparatus, like ‘open field’; others prefer to search on behavioral area, like ‘exploratory’). Note that only partial lists are shown for both interventions and methodologies.

Over 70 phenotyping and gene expression projects have been incorporated and released since our last NAR update and more are forthcoming. New data (physiology, 79%; behavior, 18%; and morphology, 3%) are classified by phenotype category and intervention/treatment, as shown in Table 2. New strain panels include HMDP, DO mice and CC lines. MPD houses pre-CC data from five projects covering a wide range of phenotypic domains: behavior, blood chemistry, hematology, body weight, body composition, exercise and endurance, response to viral infection (H1N1), kidney function, energy balance, nociception, reproductive performance and sleep patterns (14–23). We also have a growing collection of F1-diallel data from CC founder strains (15,24–26). Regarding DO data, we recently accessioned the first data set for behavioral and nociception traits (11,27). All phenotype data can be downloaded for custom analyses through the ‘Download data’ link on the homepage (left menu, Figure 1).

**Table 2.** Summary of what’s new since our last NAR update

MPD Categories with new data	
Behavior—anxiety-related	
Behavior—attentional performance	
Behavior—exploratory	
Behavior—fear conditioning	
Behavior—impulsivity	
Behavior—locomotor activity	
Behavior—reversal learning	
Behavior—stress reactivity	
Blood—clinical chemistry	
Blood—erythrocyte function	
Blood—hematology	
Blood—lipids	
Blood—serum vitamin D levels	
Body composition	
Body weight, size	
Bone mineral density	
Cancer	
Cardiovascular—blood pressure	
Cardiovascular—ECG	
Cardiovascular—heart rate	
Disease susceptibility	
Drug metabolism	
Endocrine—thyroxine	
Energy balance	
Immune system—peripheral blood lymphocytes	
Immune system—plasma immunoglobulins	
Immune system—splenocyte populations	
Ingestive preference—taste threshold (NaCl)	
Liver—function	
Liver—gene expression	
Macrophage gene expression	
Nervous system—brain morphology	
Nervous system—cell proliferation	
Nervous system—hippocampal microRNA expression	
Nervous system—infarct volume	
Nervous system—sensorimotor gating	
Nervous system—status epilepticus	
Neurosensory—eye morphology	
Neurosensory—hearing loss	
Nociception	
Pathology	
Reproduction—gestation	
Reproduction—performance	
Respiratory—lung disease	
Sleep	
Urinalysis	
Vesico-uteric reflux (newborns)	
Treatment and intervention studies with new data	
Cocaine	
DB289 (anti-parasitic drug)	
Dopamine antagonists	
Ethanol	
Nicotine	
Pilocarpine	
Aging	
Exercise	
High-fat diet	
Influenza A (H1N1)	
Influenza A (H5N1)	
*Staphylococcus aureus*	
Radiation	

### Genotype

Since our last report, the coverage of SNP and variation data (indels and structural variants) for inbred strains has increased from roughly 12–60 million genomic locations due to data released by the Wellcome Trust Sanger Mouse Genomes Project (www.sanger.ac.uk/resources/mouse/genomes/). MPD collects and integrates genotype data; this requires keeping multiple evolving annotation layers and data sets all up-to-date and in sync. Since our last NAR update, new mouse reference assembly build (GRCm38) and dbSNP annotation builds 137 and 138 have been released. MPD is currently up-to-date for all builds.

### Gene expression

MPD brings together about a dozen gene expression microarray data sets and provides some related web-based analysis tools (with several data sets and new tools added since our last report). Challenges include accommodating the mixture of platforms and annotation standards, working with static annotation files given the shifting landscape mentioned earlier in the text for genotype data, finding commonality across various complex study designs and dealing with the presence of low-sample sizes as well as other issues commonly encountered in microarray studies. To help alleviate some of these issues, we derive an overall variability metric for each probeset and provide an option that allows instances with too much variance to be omitted from results at users’ discretion.

## IMPROVEMENTS

### Web site updates

Search, navigation, layout and style updates have been applied to all areas of the web application. See Figure 1 and examples later in the text. Measurement plots and summary table formats have been updated and options reorganized. The ‘Pheno toolbox’ demo and ‘Tutorial videos’ showcase essential MPD functionality; see homepage, left menu (Figure 1). New tutorials include finding data and information about strains, explaining the suite of MPD tools, using the MPD shopping cart to collect a set of measurements of interest, getting the most out of ontologies, understanding gene expression functionality and finding genotype data and using associated tools.

### Measurement annotations and ontology implementation

All phenotype measurements in our database have had value-added annotations applied to improve the ability of researchers to find, retrieve and aggregate similar data from across diverse studies. As a result, phenotype data are now more easily located when investigating a specific intervention, phenotyping methodology or ontology term. Data and information can also be found through searches and by browsing. For example, ‘Interventions’ and ‘Methodologies’ can be browsed, as shown in Figure 1. Our interventions page has been restructured and trade name equivalents have been added for all drugs to our existing catalog of synonyms (chemicals, toxic substances, biological factors, etc). Measurements have also been annotated to a controlled vocabulary of methodology (apparatus, platform). The ‘Methodologies’ page is organized by phenotype subject area, as shown in Figure 1.

**Figure 2.**
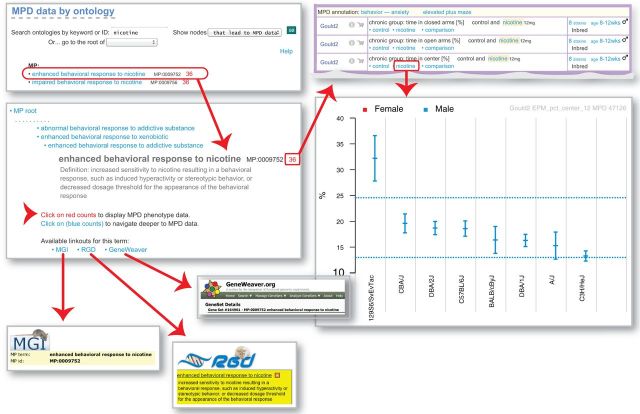
New ontology navigator and connecting to other databases (example: nicotine). The ontology browser is accessible through the ‘phenotype’ button on the homepage (Figure 1). Users enter a search term; in this example, there are two MP terms annotated to MPD measurements (upper left panel). Clicking on the term displays the definition and parent terms in the hierarchy (middle left panel). To find data annotated to this term, click on the red number as indicated by the red arrowhead; the red number shows the number of measurements directly annotated to this term. Users then get a list of available measurements to choose from (upper right panel; only a partial list is shown). Clicking on a measurement link takes users to a plot (lower right panel). In this case, 129S6/SvEvTac shows an ‘enhanced behavioral response to nicotine’. As shown in the bottom left panel, MPD provides term linkouts to MGI (31), Rat Genome Database (32) and GeneWeaver (33). This example showcases data from the new MPD project: *Gould2* (34,35).

MPD measurements have been annotated with multiple public ontologies. The Vertebrate Trait Ontology (VT) provides a standardized vocabulary to facilitate comparison of trait data within and across vertebrate species. VT is a hierarchy of terms defined as ‘measurable or observable characteristics’ related to the morphology, physiology or development of an organism (28). The Mammalian Phenotype Ontology (MP) is a tool for classifying and organizing phenotypic information associated with a mammalian species (29). The Adult Mouse Anatomical Dictionary (MA) is used to annotate and integrate data associated with mouse anatomical sites or structures (30). The use of these ontologies provides an effective way to link data from disparate sources and to facilitate convergent integration across species. MPD users can peruse the new ontology browser to locate measurements of interest, as illustrated in Figure 2. The ontology browser can be found under the ‘Phenotype’ button on the homepage (Figure 1).

### New visualization tools for Collaborative Cross lines and Diversity Outbred populations

**Figure 3.**
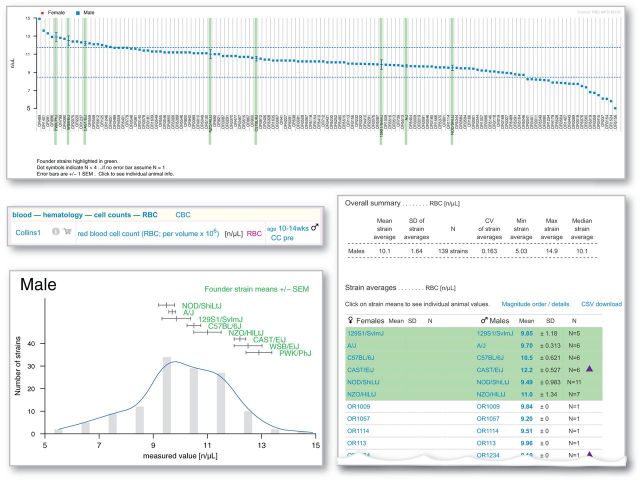
New visualization and analysis tools for Collaborative Cross data. The new CC tool plots all strains tested in a single view (upper panel), which can be wide, as in this case, where 139 strains are shown (females were not tested in this study). Founder strains are highlighted in green; overall mean and standard deviation are indicated by the horizontal dotted lines. A distribution histogram is one click away (lower left panel); for quick reference, founder strains are plotted above the histogram (mean, standard error). A data table (lower right panel, partial list) is available just below the primary plot on the Web site, showing values for strains in alphabetical order (default); up- and down-triangles indicate high- and low-end outlier strains, respectively. From here, users can opt to view data by magnitude order and view more detailed summary statistics. This example uses data from the new MPD Project: *Collins1* (16,17).

**Figure 4.**
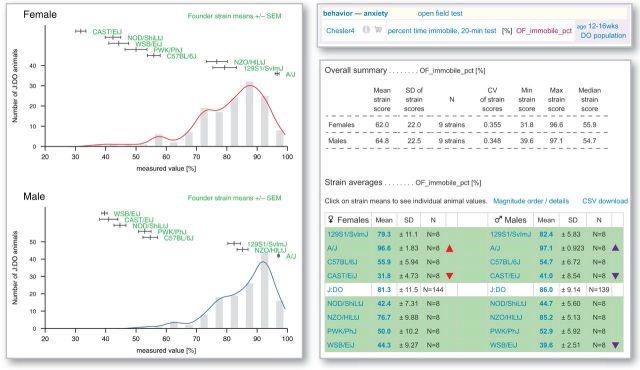
New visualization and analysis tools for Diversity Outbred populations. The new DO tool renders distribution histograms (left panel: female above, male below). Founder strains means and standard error are plotted above the histograms for quick reference. A data table is one click away (lower right panel), showing values for strains in alphabetical order (default); up- and down-triangles indicate high- and low-end outlier strains, respectively. From here, users can opt to view data by magnitude order and view more detailed summary statistics. This example uses data from new MPD Project: *Chesler4* (11,27).

We have extended functionality and developed new tools for CC lines and DO mice. The CC tool renders mean and standard error for all strains tested with founder strains highlighted, as shown in Figure 3. The ability to visualize data from all strains in a single view enables users to select optimal strains for their specific research applications. The DO tool renders a distribution histogram with founder-strain mean and standard error shown above the plot, as shown in Figure 4. For both CC and DO tools, users can access data tables and go deeper for more statistical information, including standard deviation, standard error, range, coefficient of variation and *Z*-score. Data tables may be downloaded with a single click. A phenotype characterization catalog (representing 600 + measurements) is now available for the eight CC founder strains (which are the same as DO founder strains). This functionality is accessible under the ‘Strains’ button on the homepage (Figure 1) and then through ‘Strain Panels’.

### New SNP/genotype variation query and improved gene detail pages

**Figure 5.**
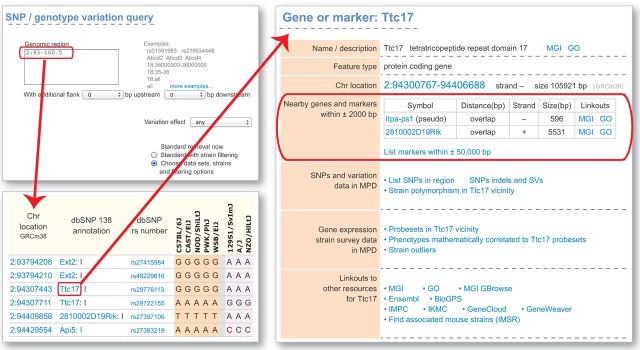
New MPD SNP query page and gene detail pages. Users must first enter genomic region (gene, marker, location or rs number; space-delimited lists can also be entered) and specify whether data sets will be manually selected and whether filtering will be implemented, e.g. polymorphisms between selected strains. The default settings use Sanger SNP data without any filtering. It is possible to ‘refine’ a query from results pages allowing users to make adjustments to the original specifications. In this example, we use results found by Logan *et al.* (11) and showcased in Figure 4 (MPD: Chesler4), where C57BL/6J, CAST/EiJ, NOD/ShiLtJ, PWK/PhJ and WSB/EiJ cluster in a group of strains that are not immobile in the open field test compared with the other CC founder strains, which cluster at the opposite end of the spectrum (129S1/SvImJ, A/J and NZO/HlLtJ). Logan *et al.* reported two QTLs for the immobility phenotype; one QTL is on chromosome 2 between 93.2–100.21 Mb. For this example, we plugged in the coordinates for this QTL and used filtering options to find polymorphic locations between the two clusters of strains (lower left panel: low immobility strains grouped to left, high immobility to right). We found four genes in this region that showed haplotypes segregating with the immobility phenotype (here showing two-color allele scheme; four-color nucleotide scheme is available as an option). Clicking on gene name takes users to the updated gene detail page. The new view shows nearby genes and markers (circled in red) with the option of viewing markers within ±50 kb (and subsequent increments). In addition, there is a link to SNPs, indels and structural variants in the region, a link to identify gene expression probesets in the vicinity and a link to find phenotypes that are correlated to those probesets. There are also convenient linkouts to other databases from gene detail pages. See text and Table 3.

We have replaced MPD’s former SNP functionality with a new genotype variation resource, as illustrated in Figure 5. A number of new data sets are included that can be combined for query. Improvements include the following new features:

- Lists of gene symbols, markers or rs numbers are supported in the query
- Flanking regions may be selected for queries involving gene symbols, markers or rs numbers
- Results tables include a column indicating distance (in bp) offset from gene symbol, marker or rs number used in the query
- Indels and structural variants can be queried and displayed in results
- SNPs and indels are linked to dbSNP rs numbers and variation-effect annotations
- Links are provided to raw data via the Wellcome Trust Sanger Web site and to dbSNP through rs number

The improved SNP/genotype variation application guides users in a multistep process (see Figure 5 legend). From the SNP results page, it is possible to collect a list of genes from the query to help narrow the list of candidates or to perform batch queries in other databases. Users can download any SNP query result. We manually fulfill investigator requests for SNP data sets that are too large to retrieve on the web (contact us phenome@jax.org).

Gene detail pages now provide more information and links to relevant content within the MPD application and to other online resources, as shown in Figure 5. One new feature is that nearby genes and markers are shown directly on gene detail pages with a one-click option to expand the view to ±50 kb and subsequent wider intervals.

## INTEGRATION WITH OTHER DATABASES

Data are imported regularly for annotation purposes: mouse strain nomenclature, genome coordinates and gene nomenclature from Mouse Genome Informatics (MGI) (32); ontology terms and structures from MGI and the VT ontology group (28); and variation-effect annotations from NCBI dbSNP (36). MPD provides convenient linkouts to several community resources. See Table 3 for a listing of Web sites that we rely on for authoritative annotations and/or to which we link; see also several examples in Figures 2 and 5. Connectivity enables bioinformatics approaches and facilitates workflows for users across platforms. MPD is also registered with the Neuroscience Information Framework (37) and is participating in a Thomson Reuters initiative to link publications and downloadable data sets (38).

**Table 3.** MPD integration and linkouts to other databases

Database	URL	References
Genome		
Mouse Genome Informatics (MGI)	informatics.jax.org	31
NCBI dbSNP	ncbi.nlm.nih.gov/SNP	36
Wellcome Trust Sanger Institute	sanger.ac.uk/resources/mouse	
Ensembl	ensembl.org	39
Rat Genome Database	rgd.mcw.edu	32
Knock-out consortia		
International Knockout Mouse Consortium (IKMC)	knockoutmouse.org	2
International Mouse Phenotyping Consortium (IPMC)	mousephenotype.org	1
Ontologies		
Mammalian Phenotype (MP)	informatics.jax.org/searches/MP_form.shtml	29
Adult Mouse Anatomy (MA)	informatics.jax.org/searches/anatdict_form.shtml	30
Gene Ontology (GO)	informatics.jax.org/searches/GO_form.shtml	40
Vertebrate Trait (VT)	bioportal.bioontology.org/ontologies/1659	28
Other Biological		
International Mouse Strain Resource (IMSR)	findmice.org	41
Mouse Tumor Biology Database (MTB)	tumor.informatics.jax.org	42
QTL Archive (Churchill Group at JAX)	qtlarchive.org	
GeneWeaver	geneweaver.org	33
BioGPS	biogps.org	43
NCBI Gene Expression Omnibus (GEO)	ncbi.nlm.nih.gov/geo	36
Pathbase	pathbase.net	
UCLA ZARLAB for EMMA analysis	whap.cs.ucla.edu/mpd	
Strains		
JAXMice	jaxmice.jax.org	
Charles River Laboratories	criver.com	
Harlan Laboratories	harlan.com	
Taconic	taconic.com	
Mouse Genetic Resource (Japan)	shigen.nig.ac.jp	
RIKEN BioResource Center (Japan)	brc.riken.jp/lab/animal	
Animal Resources Center (Australia)	arc.wa.gov.au	

## FUTURE PLANS

The original focus of MPD on a diverse set of several dozen commonly used inbred strains is still seen as valuable but over time is being superseded by other genetic reference populations, such as RI panels, chromosome substitution strains and most recently the emerging CC lines and DO mice. We will continue to collect data from these genetic reference populations. In addition, we plan to accommodate quantitative phenotype data for mutant strains. Characterization activities (phenotyping, genotyping, expression studies) are in progress by several groups in the community, and we are working to collect these data and provide analysis tools and strain–panel-specific navigation modes.

## DATA SUBMISSION

We invite investigators to submit their mouse strain data for evaluation; send inquiries to us at phenome@jax.org. Measurement descriptions and units are required along with a detailed protocol (a publication describing the experiment and procedures will suffice). Phenotyping projects are evaluated and loaded into a password-protected area for investigator preview. We work with investigators to best present their projects, and data are slated for public release soon thereafter. Note that it is not a prerequisite that a study be published in a peer-reviewed journal before posting data on the MPD Web site; however, a publication provides a valuable layer of data validation for MPD users. Contributing investigators are encouraged to mention MPD in their publications, stating that MPD is a public repository for their primary data, and to provide MPD accession numbers in their articles. See ‘Contributing data’ on the homepage, left menu (Figure 1). Contact us if you are interested in contributing data (phenome@jax.org)

## USER SUPPORT AND DEVELOPER NOTES

We have made access to the MPD help desk more visible throughout the application and continue our commitment to a one-business day response. We offer user support via email (phenome@jax.org) and provide a suggestion box (can be used anonymously); see homepage, left menu (Figure 1). Our collection of tutorials has been updated with a new set of instructional videos to help users with basic functions; see homepage, left menu (Figure 1).

## CITING MPD

For general citations of MPD or the Mouse Phenome Project, this NAR article may be referenced. In addition, the following citation format is preferred when referring to specific data sets:

Investigator(s) name (year project posted) Project title. MPD project symbol (e.g. Collins1) and/or accession number (MPD:XXX). Mouse Phenome Database Web site. The Jackson Laboratory, Bar Harbor ME, USA. URL: phenome.jax.org. Date of download or access.
